# Diagnostic Accuracy of Blood-based Biomarkers for Pancreatic Cancer: A Systematic Review and Meta-analysis

**DOI:** 10.1158/2767-9764.CRC-22-0190

**Published:** 2022-10-20

**Authors:** Laura E. Kane, Gregory S. Mellotte, Eimear Mylod, Rebecca M. O'Brien, Fiona O'Connell, Croí E. Buckley, Jennifer Arlow, Khanh Nguyen, David Mockler, Aidan D. Meade, Barbara M. Ryan, Stephen G. Maher

**Affiliations:** 1 Department of Surgery, Trinity St. James's Cancer Institute, Trinity College Dublin, Dublin, Ireland.; 2 Department of Gastroenterology, Tallaght University Hospital, Dublin, Ireland.; 3 School of Physics and Clinical Optometric Science, Technological University Dublin, Dublin, Ireland.; 4 Medical Library, Trinity College Dublin, Dublin, Ireland.

## Abstract

Pancreatic ductal adenocarcinoma (PDAC) has a 5-year survival rate below 5%. Carbohydrate antigen 19-9 (CA19-9) is the most commonly used blood-based biomarker for PDAC in current clinical practice, despite having been shown repeatedly to be inaccurate and have poor diagnostic performance. This review aims to assess the reported diagnostic accuracy of all blood-based biomarkers investigated to date in PDAC, by directly comparing individual biomarkers and multi-biomarker panels, both containing CA19-9 and not (novel). A systematic review was conducted in accordance with PRISMA standards in July 2020. Individualized search strategies for three academic databases identified 5,885 studies between the years 1973 and 2020. After two rounds of screening, 250 studies were included. Data were extracted and assessed for bias. A multivariate three-level meta-analysis with subgroup moderators was run in R using AUC values as effect size. On the basis of this model, the pooled AUC value for all multi-biomarker panels (AUC = 0.898; 95% confidence interval (CI): 0.88–0.91) was significantly higher than all single biomarkers (AUC = 0.803; 95% CI: 0.78–0.83; *P* < 0.0001). The pooled AUC value for CA19-9 alone was significantly lower compared with the multi-biomarker panels containing CA19-9 (*P* < 0.0001). For the novel biomarkers, the pooled AUC for single biomarkers was also significantly lower compared with multi-biomarker panels (*P* < 0.0001). Novel biomarkers that have been repeatedly examined across the literature, such as TIMP-1, CEA, and CA125, are highlighted as promising. These results suggest that CA19-9 may be best used as an addition to a panel of biomarkers rather than alone, and that multi-biomarker panels generate the most robust results in blood-based PDAC diagnosis.

**Significance:**

In a systematic review and three-level multivariate meta-analysis, it is shown for the first time that blood-based multi-biomarker panels for the diagnosis of PDAC exhibit superior performance in comparison with single biomarkers. CA19-9 is demonstrated to have limited utility alone, and to perform poorly in patient control cohorts of both healthy and benign individuals. Multi-biomarker panels containing CA19-9 produce the best diagnostic performance overall.

## Introduction

The search for robust biological markers for disease diagnosis and treatment has been a consistent objective within modern health care over the last few decades (1, 2). In many instances, the use of multiple biological markers is almost intuitive when proceeding with a patient diagnosis. Symptoms, for example, are biological markers of illness used to identify the ailment at hand. As with symptoms, patients will rarely experience just one, and health care professionals will generally use the presence or absence of several symptoms to make a diagnosis (3). In this way, we have used multiple biomarkers (multi-biomarker panels) for the diagnosis of disease intuitively for thousands of years. Modern medicine often overlooks the utility to be gained from the use of multi-biomarker panels, searching for single proteins or miRNAs that are dysregulated in disease and can be used for a more streamlined diagnosis. However, recent trends in the literature favor multi-biomarker panels over single biomarkers, as single biomarkers alone fail to encompass the biological complexity of disease and therefore lack the comprehensive robustness required for a confident diagnosis (2, 4, 5). This can be seen clearly in the variable diagnostic performance of certain single markers in patients with underlying conditions, and as such has caused a trend toward the use of “mixed” control cohorts, where both healthy volunteers and patients with benign conditions comprise the control cohort. In this way, the control cohort is arguably more clinically relevant, as it better represents the patient population in question.

Pancreatic cancer has one of poorest prognoses of any cancer, with a 5-year survival rate of below 5% (6). Late-stage diagnoses contribute hugely to the poor survival rates of this cancer, as the symptoms associated with pancreatic cancer can be vague in nature (7). As such, earlier diagnosis is the key to improving patient prognosis in this cancer. Pancreatic ductal adenocarcinoma (PDAC) is the most common subtype of pancreatic cancer and represents approximately 85% of all patients with pancreatic cancer (8). Currently, the only FDA-approved biomarker for PDAC diagnosis is the blood-based biomarker Carbohydrate antigen 19-9 (CA19-9; ref. 5). CA19-9 is a type of antigen released by pancreatic cancer cells, and is therefore detected at higher levels in the blood of patients with PDAC (9). However, reported sensitivity and specificity values for CA19-9 vary greatly from study to study. While sensitivity values are generally high, being reported at roughly 80%, the specificity of this biomarker has been shown to be variable, resulting in many false positives (10). Serum CA19-9 has been shown to be elevated in some benign conditions, such as pancreatitis, and also in other gastrointestinal malignancies contributing to the limitations of the biomarker (11). Furthermore, the ability to express CA19-9 at all is dependent on a patient's Lewis blood group (Le), with 5%–7% of the population belonging to the Le(a−b−) group and consequently unable to express CA19-9 at any level (12). Therefore, even though CA19-9 is widely used in current clinical practice, the results alone cannot be used to diagnose PDAC, and must always be interpreted within the clinical context of imaging and/or histopathology (13). As such, current methods of diagnosing patients with pancreatic cancer at an early stage rely heavily on the presentation of symptoms, as there is no effective method for screening patients for the presence or absence of pancreatic cancer. A recent review from our group has highlighted the importance of novel biomarker research in pancreatic patients, and argues that the integration of multiple biomarkers to form a multi-biomarker panel could be the vital next step for the identification of more robust biomarkers (5). Following on from this, we have conducted a systematic review and meta-analysis of biomarker efficacy to truly evaluate whether more really is better in the context of biomarkers.

In this systematic review, we evaluated blood-based biomarkers for the diagnosis of PDAC. The primary aim of this review is to compare the efficacy of single biomarkers and multi-biomarker panels in the context of PDAC diagnosis to determine which biomarker type performs better. The secondary objective is to examine the current clinical standard, CA19-9, and its performance comparatively to novel biomarkers for PDAC diagnosis. The final objective of this review is to highlight promising novel biomarkers that have been examined repeatedly in the literature and may provide direction for future biomarker studies.

## Materials and Methods

A systematic review of blood-based biomarkers for the diagnosis of PDAC was conducted in accordance with PRISMA standards (Supplementary Material S1 and S2; ref. 14). The review was registered with PROSPERO prior to data extraction (CRD42020207241).

### Search Strategy and Inclusion Criteria

Academic databases MEDLINE, Web of Science, and EMBASE were searched using individualized search strategies containing both medical subject headings and text words (Supplementary Material S3). Publications were limited to those written in English, conducted in human participants and published on or before July 20th, 2020. No limit was placed on the date of publication prior to the date of the literature search. Studies that were identified were exported to Endnote X9 and subsequently imported to www.covidence.org for review, where covidence analytics removed any duplicates (Supplementary Material S2).

Human studies reporting on blood-based single biomarkers or multi-biomarker panels for the diagnosis of PDAC were included. As PDAC is considered to be synonymous with pancreatic cancer, where a study did not specify the subtype of pancreatic cancer, it was assumed that this study was referring to PDAC and it was therefore included in the review. Only studies examining primary PDAC in patients of any stage, with or without metastasis, were included. Both PDAC and control cohorts must have had a minimum of 15 patients to be included in the study. This was to ensure sufficient statistical power in each study according to power calculations (15). Studies reporting on image-based diagnostic methods, such as endoscopic ultrasounds or CT scans, or pancreatic cyst fluid and/or pancreatic tissue-based biomarkers were excluded as they were not deemed directly comparable with blood-based biomarkers. Studies reporting on biomarkers of any omic compartment, for example, proteomics, transcriptomics, or genomics, were included. Multi-biomarker panels consisting of biomarkers from different omic compartments were also included. Studies where the patient data were obtained from an online database were excluded to avoid examining biomarkers that were assessed in the same patient cohorts. Where all patients in a study, PDAC and controls, had preexisting conditions, this study was excluded. Only studies which reported a significant result were included to ensure the results did not skew the downstream analyses. A full list of inclusion and exclusion criteria is given in Supplementary Material S4.

Title and abstract screening was conducted independently by two randomly assigned reviewers, and included studies were then subject to full-text screening in the same manner. Any disagreements were discussed and settled by two senior reviewers (L.E. Kane and G.S. Mellotte). Where the full-text of an article could not be located, corresponding authors were contacted to request access to the article. Reasons for exclusion at the full-text stage are given in Supplementary Material S2.

### Data Extraction and Risk of Bias Assessment

An extraction template in Excel was piloted by two reviewers for a small subset of papers before being finalized. Reviewers extracted data into their own preoptimized template in Excel, with data compilation being carried out once all studies had been extracted. Data extracted included information such as study details (title, corresponding author name and email address, country of study, dates conducted); biomarker details (biomarker name(s), biological properties, detection platform); patient cohort details (number patients per cohort, sex and age breakdown, condition); and reported statistics (analysis performed, *P* value, sensitivity, specificity, AUC). A complete list of extracted data fields is included in Supplementary Material S5. Data were extracted such that each row represented an individual biomarker or multi-biomarker panel having been assessed in one set of patient cohorts. Where a biomarker or multi-biomarker panel was assessed in multiple cohorts, for example, training and validation cohorts, these data were extracted into separate rows. As a result, larger studies which examined multiple biomarkers in multiple patient cohorts, represent more rows of data than smaller studies.

All included studies were assessed for quality and risk of bias (RoB) using the QUADAS-2 tool. The QUADAS-2 tool provides an assessment for the level of bias an individual study will introduce into the systematic review based on the nature of its design. The selected questions from the four main domains of the tool were amended to align with the review, as per the QUADAS-2 guidelines. This assessment was carried out by reviewers in tandem with the data extraction. Responses were given as either “yes,” “no”, or “unclear” and domains were subsequently scored as “high,” “low”, or “unclear” RoB. Selected questions for the RoB assessment are included in Supplementary Material S6.

Given the high volume of included papers, studies were only extracted and RoB assessed by a single reviewer. To assess the accuracy of this process a random selection of 25 papers, to represent 10% of the total number of studies included, was generated using R. These papers were extracted and RoB assessed by a second reviewer, with both data extractions and RoB assessments subsequently checked for mistakes and/or missing information by a senior reviewer (L.E. Kane or G.S. Mellotte). Extraction accuracy was then calculated for each paper using the total number of correct datapoints as a percentage of the total number of datapoints. If there were disagreements between both senior reviewers with regards to the eligibility of a study, or a discrepancy of greater than 10% for the accuracy of the RoB or data extraction, a third reviewer was consulted (S.G. Maher) to settle disputes. An Excel file with all extracted biomarker and cohort details are given in Supplementary Material S7.

### Statistical Analysis

Data filtering and clean-up was conducted in Microsoft Excel. To calculate uniform 95% confidence intervals (CI) for reported sensitivity and specificity values, 2 × 2 contingency tables were constructed using the extracted values for sensitivity, specificity, number of patients with PDAC, and number of control patients. Both two-level and three-level meta-analyses were run on the data to identify the model of best fit. The three-level model had significantly lower Akaike Information Criterion (AIC) and Bayesian Information Criterion (BIC) values, and was therefore deemed to be the most appropriate model (*P* < 0.0001). A multivariate three-level meta-analysis with subgroup moderators was run in R (v 1.3.959) with the “metaphor” package (v. 3.0-2) using reported AUC values as effect size (16, 17). AUC values <0.5 were removed from the analysis as they are regarded as diagnostically useless. Studies where PDAC was not specified (*n* = 25) were excluded from the primary meta-analysis. A secondary meta-analysis, including all 250 studies, was also conducted. Figures were created in GraphPad Prism v9.2.0 and Microsoft PowerPoint v2108.

### Patient and Public Involvement

Given the nature of this systematic review and meta-analysis, it was not possible to involve patients or the public in this research.

### Data Availability

The data generated in this study are available in Supplementary Material S7. The methodology utilized to conduct this review has been registered with, and can be viewed on, PROSPERO (CRD42020207241).

## Results

### Identification of Relevant Studies

After removing duplicates, 5,885 studies were identified by our literature search as potential candidates for inclusion in this review. After two stages of screening by reviewers, 250 papers were included in this review (Supplementary Material S2). Most records excluded at the full-text stage were omitted due to having cohorts of less than 15 patients, no accessible full-text, or being a conference proceeding and therefore not a peer-reviewed full-text paper.

### Accuracy and RoB Assessment

**FIGURE 1:**
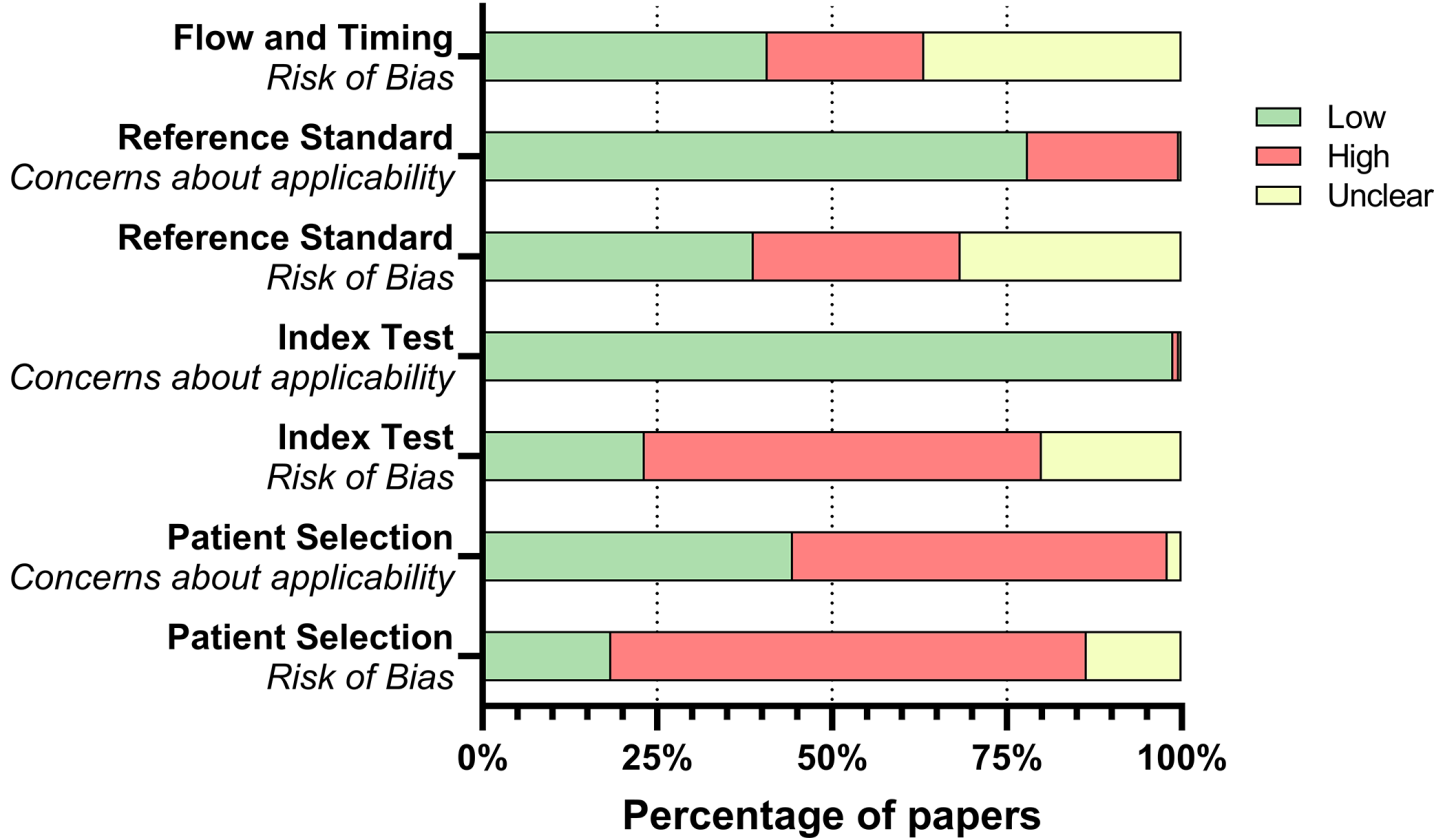
Summary of results for the QUADAS-2 RoB and study quality assessment.

Summarized results for RoB and quality assessment as conducted through the use of the QUADAS-2 tool are shown in Figure 1. Concerns regarding index test applicability were generally low once a blood-based biomarker was being assessed for PDAC diagnosis as per the inclusion criteria of the review. Patient selection was frequently high risk (68%) as control cohorts were often not clinically relevant, that is, contained healthy or benign patients only. RoB was low for reference standards (38.8%) and index tests (23.2%) when studies were blinded to the results, and unclear (31.6% and 20%, respectively) when no details were given. Concerns about the applicability of the index test were low in most cases (98.8%) as most biomarkers were for the diagnosis of PDAC.

The accuracy of data extraction and RoB assessments were independently assessed by senior reviewers before proceeding to the data analysis stage. Data extraction was shown to have a mean accuracy of 91.47% (95% CI: 91.42–91.52), and QUADAS-2 RoB assessment was shown to have a mean accuracy of 92.63% (95% CI: 92.59–92.67).

### Summary of Extracted Studies

The data extracted from the 250 papers included in the study are broadly summarized in Table 1. As stated previously, CA19-9 is the current FDA-approved biomarker for PDAC diagnosis. However, only 51.6% of papers included this biomarker in their study, while 96% of papers evaluated novel biomarkers. A total of 2,077 rows of data were extracted, each representing an individual biomarker entry, with 982 distinct biomarkers included in the analysis. All studies examined blood-based biomarkers in either plasma, serum, or whole blood, with the majority of entries (67.9%) being investigated in serum. While 79.9% of studies recruited patients prospectively, 12.5% retrospectively examined clinical data of patients with PDAC from a hospital database within a certain time period (mean = 3.7 years, range = 14–0.6 years), and the remaining 7.6% were unclear about the recruitment process of patients. Study size varied greatly between papers, with PDAC cohorts ranging from 15 to 809 patients, and control cohorts ranging from 15 to 898 patients. Blinding across studies was shown to be poor, with only 35.6% of entries examined under blinded conditions, and 15.6% unclear on whether the study was blinded or not. As PDAC is synonymous with pancreatic cancer, studies that did not include a subtype of pancreatic cancer were assumed to be PDAC. Importantly, for 183 biomarker entries (8.8%) there was no specific subtype of pancreatic cancer given. PDAC diagnosis by use of a given reference standard (e.g., histology, cytology) was reported in most cases (55.8%). However, 44.2% of biomarker entries reported no reference standard for PDAC diagnosis. Furthermore, 29.4% provided no sex breakdown, 37.2% gave no indication of the age demographics, and 31.3% of entries had no information regarding the stage of patients with PDAC. Similarly, a substantial number of entries had no information on patient sex (32.2%) or age (42.5%) for their control cohorts. Qualitative assessments of biomarker efficacy were provided for most entries (71.2%); however, 598 entries (28.9%) contained only a *P* value and did not provide any sensitivity, specificity or AUC values. More than 40% of biomarkers had no AUC value and were therefore not included in the meta-analysis.

**TABLE 1:** Summary of extracted papers

**Summary of papers**			
***Total extracted*** ^a^	**250**	***PDAC cohort details***	
***Examining >1 biomarker***	**196** (78.4%)	Mean PDAC cohort size (range)	**60.28** (15–809)
***Examining CA19* *-* *9 biomarkers***	**129** (51.6%)	PDAC reference standard	**1,158** (55.8%)
***Examining novel biomarkers***	**240** (96%)	No PDAC reference standard	**919** (44.2%)
		No PDAC stage details	**650** (31.3%)
**Summary of unique biomarker entries**		No sex breakdown	**611** (29.4%)
***Total number of biomarker entries*** ^b^	**2,077**	No age mean/median/range	**773** (37.2%)
Novel (Single/Multi)	**1,467** (1,228/239)	***Control cohort details***	
CA19-9 (Single/Multi)	**610** (293/317)	PDAC vs. healthy^c^	**1,084** (52.2%)
***Number of unique biomarkers***	**982**	*Mean cohort size (range)*	**50.7** (15–898)
Novel (Single/Multi)	**815** (675/140)	*No sex breakdown*	**356** (32.8%)
CA19–9 (Single/Multi)	**167** (1/166)	*No age mean/median/range*	**460** (42.4%)
***Fluid type***		PDAC vs. benign^d^	**867** (41.7%)
Serum	**1,411** (67.9%)	*Mean cohort size (range)*	**42.47** (15–786)
Plasma	**576** (27.7%)	*No sex breakdown*	**281** (32.4%)
Whole Blood	**88** (4.2%)	*No age mean/median/range*	**393** (45.3&)
Serum and Plasma	**2** (0.09%)	PDAC vs. mixed^e^	**126** (6.1%)
***Study design***		*Mean cohort size (range)*	**71.78** (33–199)
Prospective	**1,659** (79.9%)	*No sex breakdown*	**31** (24.6%)
Retrospective	**259** (12.5%)	*No age mean/median/range*	**30 (23.8%)**
Unclear	**159** (7.6%)	Total number of entries with:	
***Cancer type***		*No sex breakdown*	**668** (32.2%)
PDAC specified	**1,894** (91.2%)	*No age mean/median/range*	**883** (42.5%)
PC unspecified	**183** (8.8%)	***Statistical analyses***	
***Cohort blinding***		Qualitative assessment	**1,479** (71.2%)
Blinded	**740** (35.6%)	*P*-value alone	**598** (28.9%)
Unblinded	**1,012** (48.7%)	AUC	**1,206 (**58.1%)
Unclear	**325** (15.6%)	Sensitivity/Specificity	**900 (**43.3%)

NOTE: Percentages for the “Summary of papers” use denominator^a^. Percentages for the “Summary of unique biomarker entries" use denominator^b^, except where patient control cohorts are broken down and percentages use denominators^c–e^.

### Meta-analysis: Full Dataset

**FIGURE 2:**
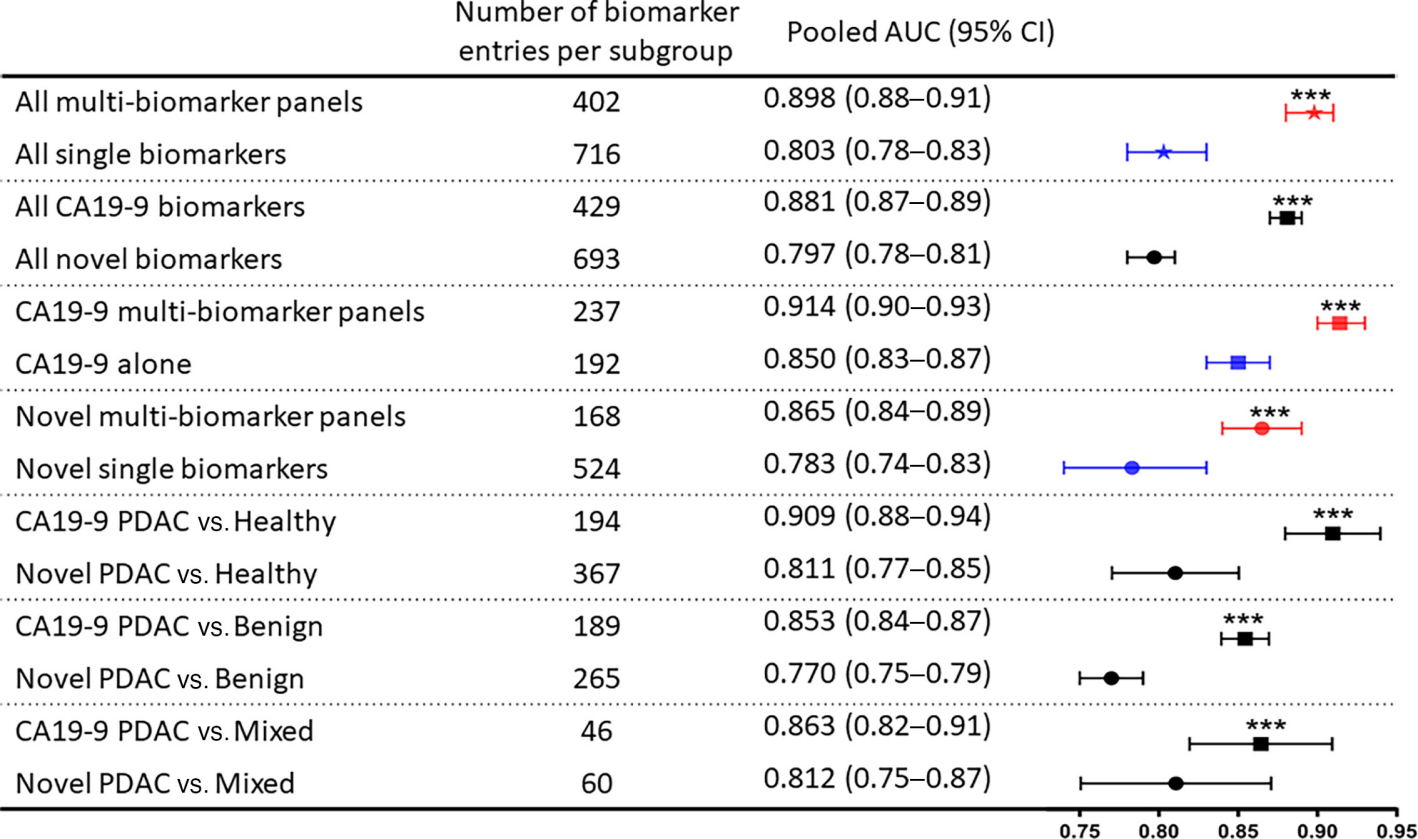
Summary of multivariate three-level meta-analysis with subgroup moderators. Number of biomarker entries for each subgroup are given. The forest plot shows the pooled AUC value and 95% CIs for each biomarker subgroup from the multivariate three-level meta-analysis. Subgroups directly compared are separated by a dotted line. Symbols represent the whole dataset (★), CA19-9 subgroup (■) and novel subgroup (●). Colors represent biomarker type: all types (black), multi-biomarker panels (red), and single biomarkers (blue). Significantly higher AUC values are denoted using asterisks. ***, *P* < 0.0001.

**FIGURE 3:**
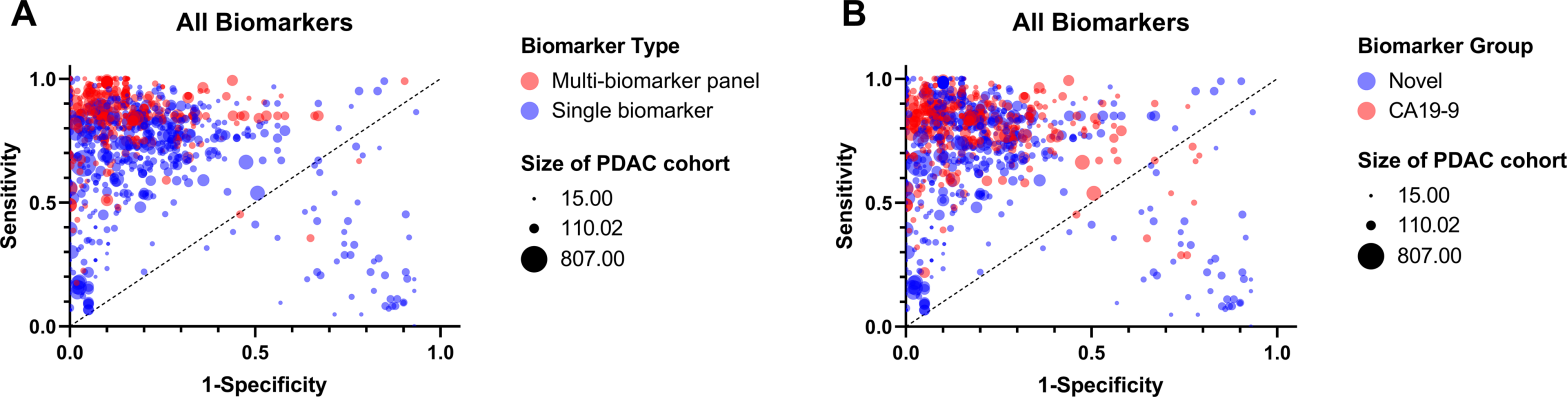
Comparison of single biomarkers and multi-biomarker panels overall and subdivided by biomarker group. Representative ROC plot showing the extracted sensitivity and 1-specificity for all biomarkers. Symbol colors represent biomarker type (**A**) and biomarker group (**B**), while symbol size represents the number of patients in the PDAC cohort for the given statistics.

On the basis of the multivariate three-level meta-analysis with subgroup moderators, the pooled AUC value for all multi-biomarker panels (AUC = 0.898; 95% CI: 0.88–0.91) was significantly higher compared with single biomarkers (AUC = 0.803; 95% CI:0.78–0.83; *P* < 0.0001; Fig. 2). Overall, multi-biomarker panels show improved sensitivity and specificity compared with single biomarkers (Fig. 3A). To further interrogate these data, biomarkers were subdivided into two groups: those including the current standard biomarker for pancreatic patients, CA19-9, and those without (herein known as novel biomarkers). The pooled AUC value for CA19-9–containing biomarkers (AUC = 0.881; 95% CI: 0.87–0.89) was significantly higher compared with novel biomarkers (AUC = 0.797; 95% CI: 78–81; *P* < 0.0001). The sensitivity and specificity values for CA19-9 biomarkers appear improved compared with novel biomarkers (Fig. 3B). A second meta-analysis was also conducted, including all studies, even those for which PDAC is not specified. There was no notable difference between the results of these two meta-analyses (Supplemen-tary Material S8A).

### Meta-analysis: CA19-9 and Novel Biomarker Subgroups

**FIGURE 4:**
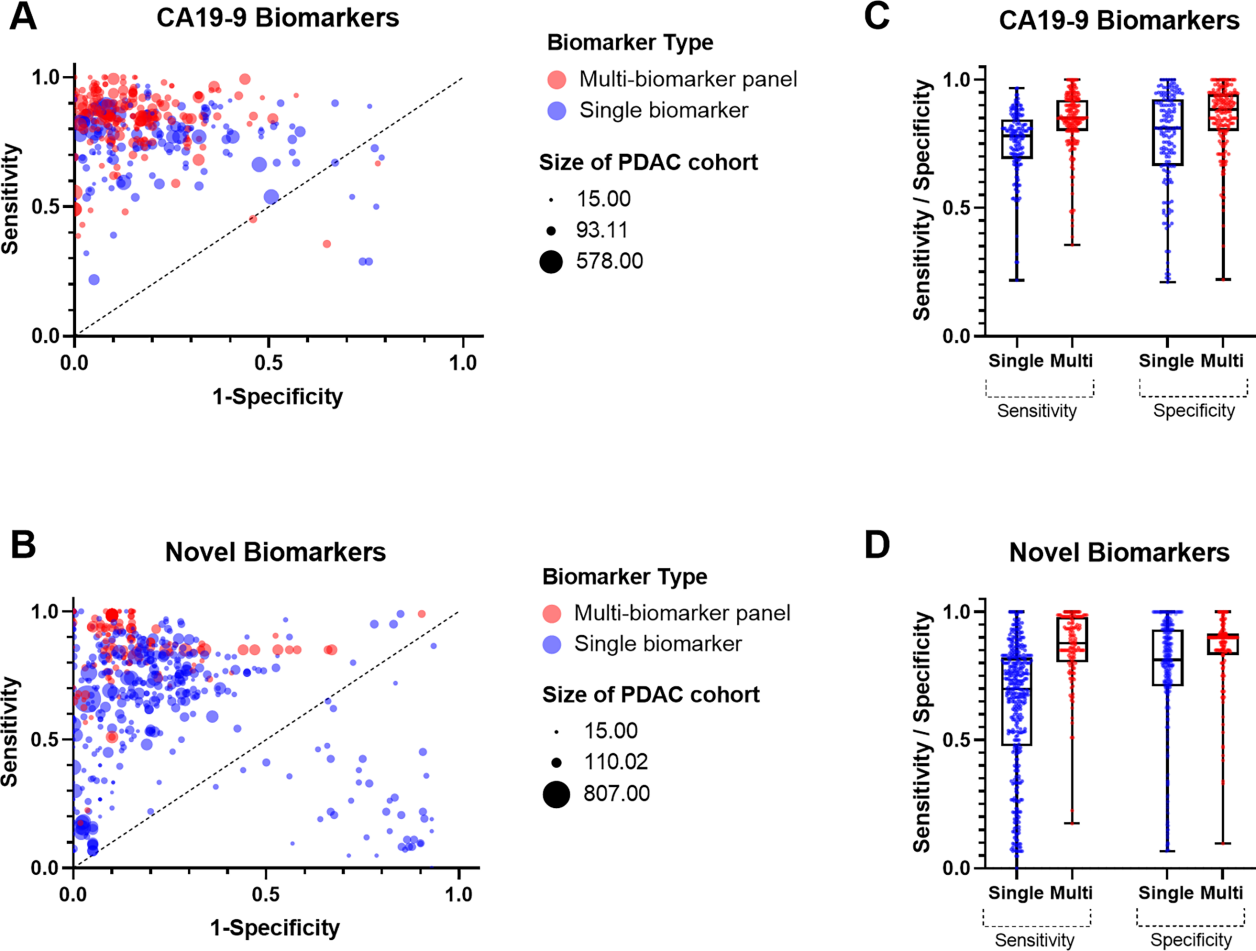
Comparison of single biomarkers with multi-biomarker panels for both CA19-9 and novel cohorts. Representative ROC plot showing the extracted sensitivity and 1-specificity for all biomarkers containing CA19-9 (**A**) and all novel biomarkers (**B**). Symbol colors represent biomarker type while symbol size represents the number of patients in the PDAC cohort for the given statistics. Box and whisker plot showing the extracted sensitivity and specificity of all biomarkers containing CA19-9 (**C**) and all novel biomarkers (**D**). Individual datapoints are represented by the colored dots over the plot. Whiskers show the maximum and minimum value, boxes show the 25th and 75th percentiles, and the line within the box indicates the median.

Examining the CA19-9 and novel subgroups independently, the pooled AUC value for CA19-9 alone (AUC = 0.85; 95% CI: 0.83–0.87) was significantly lower compared with the multi-biomarker panels containing CA19-9 (AUC = 0.914; 95% CI: 0.90–0.93; *P* < 0.0001; Fig. 2). Multi-biomarker panels containing CA19-9 have improved sensitivity and specificity when compared with CA19-9 alone (Fig. 4A). There is a large amount of variation in the reported sensitivity and specificity values between studies for the current standard biomarker, CA19-9, with some papers reporting values that fall below the random classifier line on the ROC plot. The estimated between-study variance in the model was *I*^2^_Level 3_ = 64.49%, and the within-study variance was *I*^2^_Level 2_ = 35.51%. We noted that this variation in CA19-9 was not a result of platform-to-platform discrepancies in CA19-9 detection throughout the studies examined in this review, as both immunoassays and mass-spectrometry-based detection of CA19-9 showed high variation within their respective platforms (Supplementary Material S8B). As such, the differences observed are more likely to be a result of the patient populations examined rather than the platforms used. For the novel biomarkers, the pooled AUC for single biomarkers (AUC = 0.783; 95% CI: 0.74–0.83) was also significantly lower compared to novel multi-biomarker panels (AUC = 0.865; 95% CI: 0.84–0.89; *P* < 0.0001). Novel multi-biomarker panels show improved sensitivity and specificity values over single biomarkers alone (Fig. 4B and D). Furthermore, there is less variation in the sensitivity and specificity values for multi-biomarker panels containing CA19-9 than CA19-9 alone, with a smaller interquartile range and higher mean and median values being shown for both test statistics (Fig. 4C).

### Meta-analysis: CA19-9 and Novel Biomarkers in Different Patient Cohort Subgroups

**FIGURE 5:**
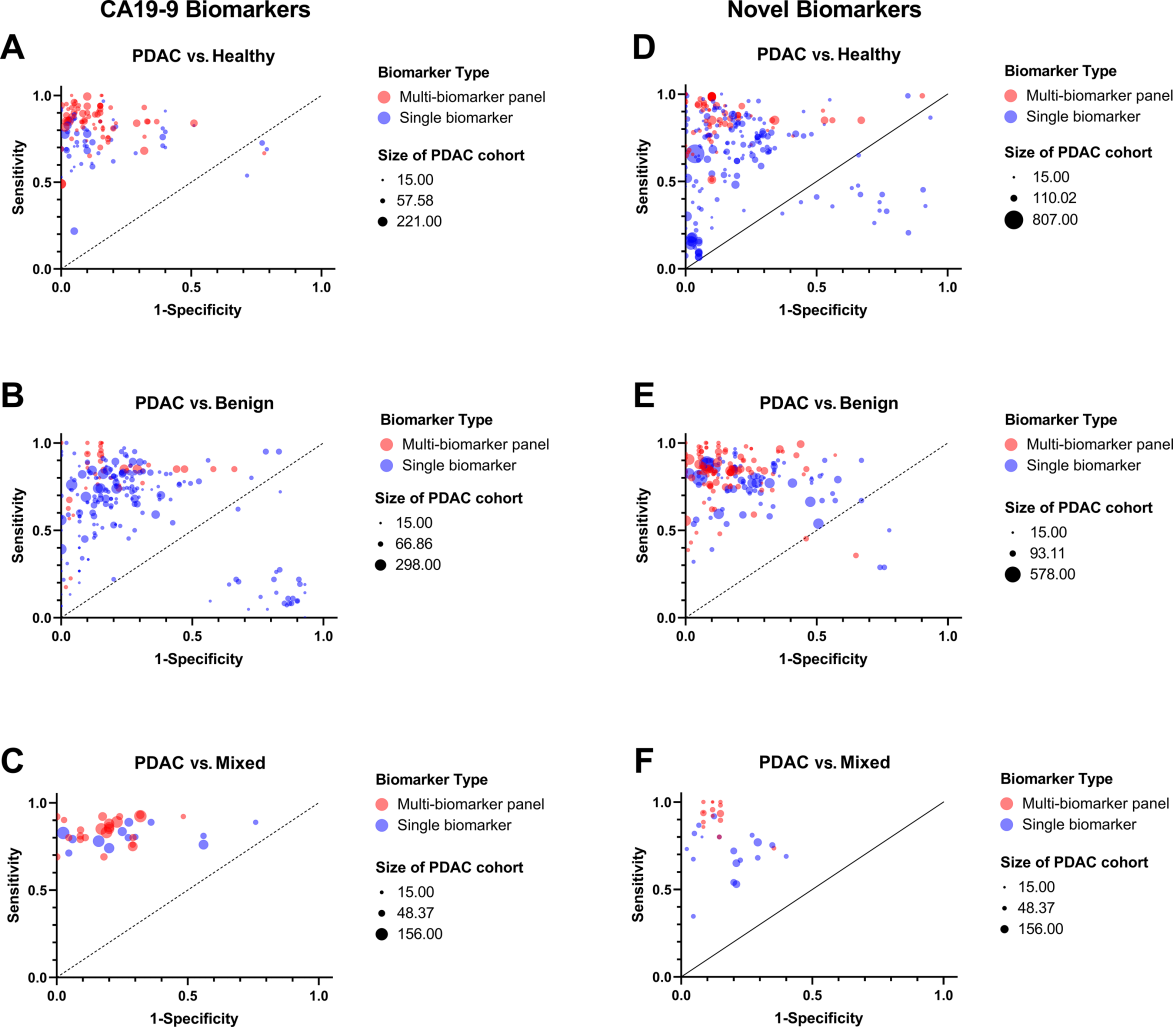
Comparison of CA19-9 and novel biomarkers subdivided by patient cohorts. Representative ROC plots showing the extracted sensitivity and 1-specificity for all biomarkers. Comparison of CA19-9 alone (blue) and multi-biomarker panels (red) for PDAC versus healthy (**A**), PDAC versus benign (**B**), and PDAC versus mixed patient (**C**) cohorts. Comparison of novel single biomarkers (blue) and novel multi-biomarker panels (red) for PDAC versus healthy (**D**), PDAC versus benign (**E**), and PDAC versus mixed patient (**F**) cohorts. Symbol size presents the number of patients in the PDAC cohort for the given statistics.

To further evaluate the efficacy of each biomarker and/or panel, results were subdivided on the basis of the patient cohorts involved as follows: PDAC versus healthy, PDAC versus benign, and PDAC versus mixed (healthy and benign; Fig. 5). Multi-biomarker panels demonstrate improved sensitivity and specificity when compared with single biomarkers across all patient cohorts. On the basis of the meta-analysis, biomarker robustness was also influenced by the patient cohort examined, with CA19-9–containing biomarkers performing best in all cohorts compared with novel biomarkers: PDAC versus healthy (AUC = 0.909; 95% CI: 0.88–0.94), PDAC versus benign (AUC = 0.853; 95% CI: 0.84–0.87), and PDAC versus mixed (AUC = 0.863; 95% CI: 0.82–0.91; *P* < 0.0001; Fig. 2). Furthermore, CA19-9 biomarkers examined in PDAC versus healthy cohorts have improved AUC values compared with those examined in PDAC versus mixed cohorts (*P* < 0.0001).

### Biomarker Efficacy of Different “omics” Compartments

Proteomic biomarkers are the most frequently evaluated blood-based biomarkers for pancreatic cancer diagnosis (Supplementary Material S8C). Proteomic biomarkers represent 77.9% of novel single biomarkers and are present in 50.3% of novel multi-biomarker panels examined. Of the panels where some other biomarker(s) was combined with the current standard, CA19-9, 76.4% opted to add another protein biomarker. Given the lack of diversity in omic compartments between studies, no distinct difference can be observed between the sensitivity and specificity values for biomarkers of different omic compartments.

Given the high prevalence of proteomic-orientated studies, biomarkers were pooled into categories that represent generalized cell compartments as follows: Genomics & Transcriptomics & Epigenomics; Metabolomics & Proteomics; and Single-cell omics & Immunomics. After pooling biomarkers into these groups, there was still no visible difference in sensitivity or specificity values between the different omic compartments (Supplementary Material S8D).

### Biomarkers in the 90th Percentile for Sensitivity and Specificity

**FIGURE 6:**
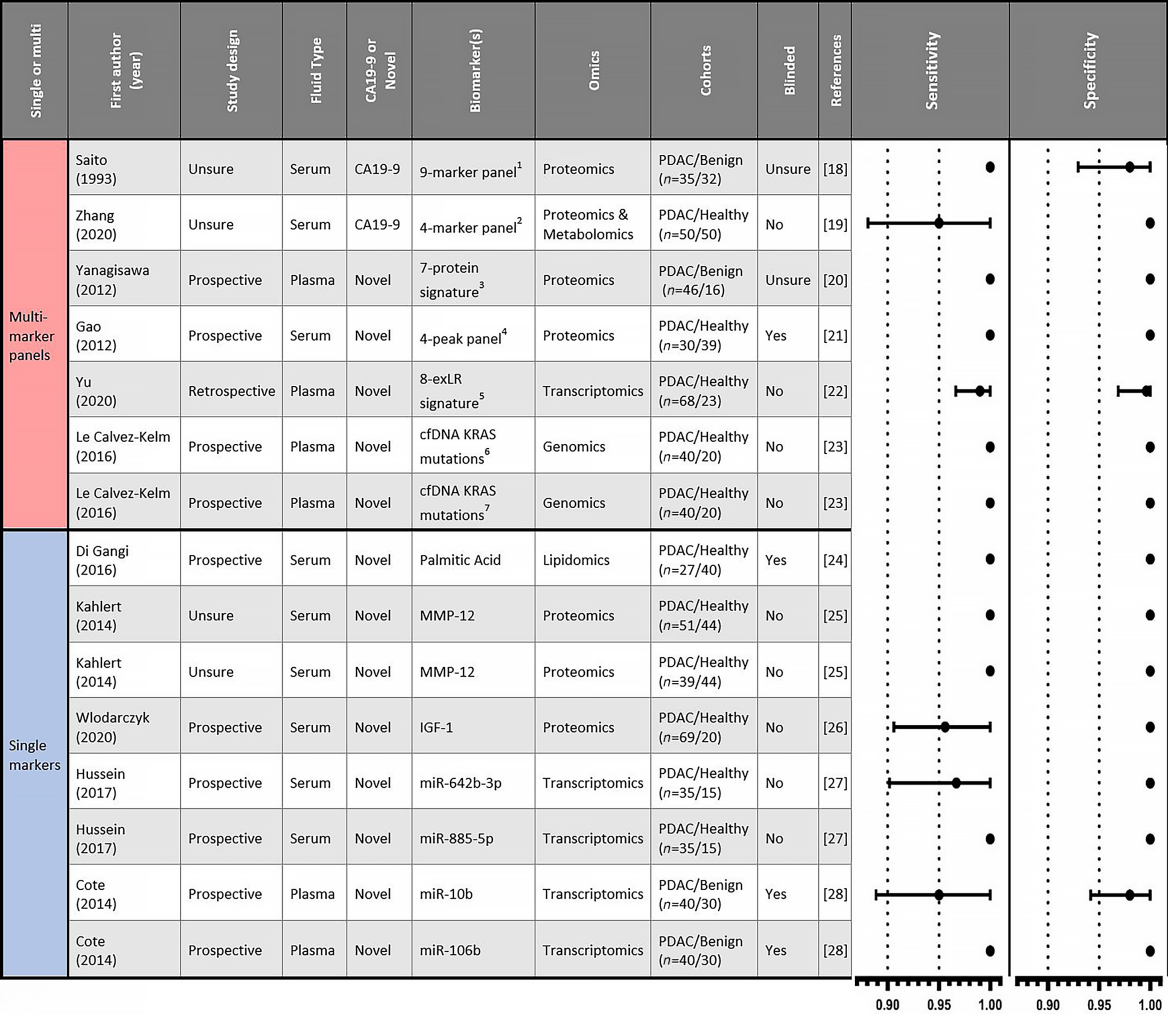
Details of the biomarkers in the 90th percentile for sensitivity and specificity. Details of the 15 biomarkers that are in the 90th percentile of all biomarkers for both sensitivity (≥0.95) and specificity (≥0.979). Forest plots of sensitivity and specificity values with 95% CIs for each biomarker are shown. ^1^CA19-9, DUPAN-2, TPA, elastase-1, lipase, amylase, gamma-glutamyl transpeptidase, alkaline phosphatase, and lactate dehydrogenase. ^2^CA19-9, docosahexanoic acid, lysoPC(14:0), and histidinyl-lysine. ^3^8562.3m/z, 8684.4m/z, 8765.1m/z, 9423.5m/z, 13761.5m/z, 14145.2m/z, and 17250.8m/z. ^4^7,775 Da, 8,567 Da, 5,362 Da, and 5,344 Da. ^5^FGA, KRT19, HIST1H2BK, ITIH2, MARCH2, CLDN1, MAL2, and TIMP1. ^6^cfDNA KRAS mutations at PDAC hotspot codons (12, 13, 61). ^7^cfDNA KRAS mutations at any screened codons reported in any cancer sites.

Biomarkers in the 90th percentile of all entries for which there are sensitivity and specificity values, represented by a sensitivity value equal to or above 0.95 and a specificity value equal to or above 0.979, are summarized in Figure 6 (18–28). A total of 15 biomarkers comprise the 90th percentile, with seven of those being multi-biomarker panels. Most of these biomarkers were proteomic (*n* = 6) or transcriptomic (*n* = 5), with just one representing more than one omic compartment (proteomics and metabolomics). These biomarkers were identified from just 11 studies spanning 27 years (1993–2020), with four studies reporting on two biomarkers each. Nine of the top 15 biomarkers reported perfect sensitivity (1.00) and specificity (1.00), with a 95% CI of ± 0. Nine biomarkers are reported from studies that were not blinded, while just four biomarkers out of the 15 biomarkers were examined in a blinded study design. The most common patient cohorts for biomarker assessment were PDAC versus healthy (*n* = 11), with none of the top 15 biomarkers having been examined in the more clinically relevant PDAC versus mixed cohort. This is reflected in the RoB assessments for these studies, where several have high levels of bias for patient selection and index test (Supplementary Material S8E). Only two biomarkers contained the current clinical standard biomarker, CA19-9, with 13 of 15 biomarkers being novel biomarkers. Furthermore, both biomarkers in the CA19-9 subgroup were multi-biomarker panels, with no reported sensitivity or specificity values for CA19-9 alone across all 250 included papers being within the 90th percentile of examined biomarkers.

### Most Frequently Examined Novel Biomarkers Across Included Studies

Novel biomarkers that have been examined most frequently across the studies included in this review are shown in Table 2. A total of 13 novel biomarkers were examined in more than one study and had a minimum of 20 unique entries. Tissue inhibitor matrix metalloproteinase 1 (TIMP-1) is the most examined biomarker, appearing 79 times in 10 studies, with CEA being a close second with 73 unique appearances in 34 studies. MiR-21 had the lowest number of unique appearances at 21, though these spanned across 10 studies. Of these 13 novel biomarkers, 10 were proteomic and three were transcriptomic in nature, with all 13 having been examined both alone and as part of a panel. The mean sensitivity of all novel biomarkers is higher when examined as part of a multi-biomarker panel. This holds true for mean specificity in most biomarkers also, with just CA125 and thrombospondin-2 (THBS2) showing improved mean specificity alone compared with when part of a panel. Mean AUC values are higher when examined as part of a panel for all biomarkers except Mucin 5AC (MUC5AC), with superior mean AUC values for this biomarker being found when examined alone. Albumin (ALB) had the highest mean sensitivity values both alone and as part of a panel, with LRG1 having the lowest in both cases. THBS1 had the highest mean specificity both alone and as part of a panel, with LRG1 again performing the worst in both categories. MUC5AC had the highest mean AUC value alone; however, it had the lowest mean AUC value as part of a panel. Conversely, ALB has the highest mean AUC value as part of a panel, and the lowest mean AUC value when examined alone. TIMP-1 is the only of these biomarkers that also appears among the biomarkers in the 90th percentile in Figure 6, where it appears as part of an 8-biomarker panel.

**TABLE 2:** Performance of the most frequently examined novel biomarkers

Novel biomarker	Number of papers	Omic compartment	Number of unique appearances		Sensitivity range (mean)	Specificity range (mean)	AUC range (mean)
**TIMP-1**	**10**	**Transcriptomics**	**79**	*Alone: 30*	0.1–0.5 (0.28)	0.09–0.96 (0.4)	0.61–0.95 (0.78)
				*Part of panel: 49*	0.36–1 (0.89)	0.22–1 (0.81)	0.83–0.99 (0.94)
**CEA**	**34**	**Proteomics**	**73**	*Alone: 42*	0.06–0.8 (0.41)	0.17–0.99 (0.68)	0.53–0.82 (0.67)
				*Part of panel: 31*	0.16–0.98 (0.77)	0.67–1 (0.9)	0.78–0.99 (0.92)
**CA242**	**5**	**Proteomics**	**45**	*Alone: 19*	0.31–0.83 (0.62)	0.51–1 (0.78)	0.62–0.89 (0.75)
				*Part of panel: 26*	0.57–0.97 (0.77)	0.75–1 (0.92)	0.8–0.98 (0.9)
**CA125**	**11**	**Proteomics**	**38**	*Alone: 20*	0.07–0.89 (0.42)	0.69–1 (0.93)	0.57–0.79 (0.7)
				*Part of panel: 18*	0.79–0.99 (0.96)	0.69–096 (0.89)	0.8–0.93 (0.87)
**miR-483**	**2**	**Transcriptomics**	**37**	*Alone: 8*	N/A	N/A	0.7–0.75 (0.73)
				*Part of panel: 29*	N/A	N/A	0.63–0.99 (0.82)
**MUC5AC**	**3**	**Proteomics**	**31**	*Alone: 27*	0.65–0.95 (0.78)	0.7–0.9 (0.77)	0.68–0.94 (0.82)
				*Part of panel: 4*	N/A	N/A	0.69–0.93 (0.81)
**THBS2**	**5**	**Proteomics**	**28**	*Alone: 15*	0.07–0.52 (0.19)	0.974–1 (0.99)	0.61–0.89 (0.79)
				*Part of panel: 13*	0.62–0.9 (0.75)	0.9–1 (0.95)	0.76–0.98 (0.9)
**IL-8**	**7**	**Proteomics**	**24**	*Alone: 6*	0.15–0.72 (0.33)	0.72–0.95 (0.83)	0.6–0.71 (0.65)
				*Part of panel: 18*	0.75–0.99 (0.87)	0.58–1 (0.85)	0.81–1 (0.92)
**CRP**	**5**	**Proteomics**	**24**	*Alone: 7*	0.2–0.77 (0.48)	0.55–0.93 (0.71)	N/A
				*Part of panel: 17*	0.83–0.99 (0.96)	0.9–1 (0.91)	0.91–0.98 (0.96)
**ALB**	**4**	**Proteomics**	**24**	*Alone: 4*	0.79–0.79 (0.79)	N/A	0.18–0.87 (0.4)
				*Part of panel: 20*	0.92–0.99 (0.97)	0.9–1 (0.91)	0.95–0.98 (0.97)
**LAMC2**	**2**	**Proteomics**	**23**	*Alone: 11*	N/A	N/A	0.65–0.87 (0.81)
				*Part of panel: 12*	N/A	N/A	0.8–0.96 (0.88)
**LRG1**	**3**	**Proteomics**	**22**	*Alone: 10*	0.5–0.46 (0.17)	0.1–0.37 (0.19)	0.64–0.94 (0.77)
				*Part of panel: 12*	0.36–0.92 (0.72)	0.22–0.83 (0.56)	0.82–0.96 (0.89)
**miR-21**	**10**	**Transcriptomics**	**21**	*Alone: 17*	N/A	N/A	0.49–0.99 (0.7)
				*Part of panel: 4*	0.85–0.9 (0.87)	0.85–0.87 (0.86)	0.82–0.95 (0.88)

NOTE: Sensitivity, specificity, and AUC value breakdowns are given for novel biomarkers that were examined in more than one study and had a minimum of 20 unique entries. N/A indicates that there are no extracted data for this field for a minimum of two biomarker entries.

## Discussion

Currently, there is no biomarker that can effectively and consistently discriminate patients with PDAC from those without. The aim of this systematic review and meta-analysis was to examine the performance of all published blood-based biomarkers used for the diagnosis of PDAC. Specifically, we examined papers that evaluated some blood-based biomarker(s) for the diagnosis of PDAC, with no limit placed on the publication date of the paper or the “omic” compartment of the biomarker(s) assessed. We evaluated whether single biomarkers or multi-biomarker panels generally have the best efficacy for PDAC diagnosis by performing a multivariate three-level meta-analysis using AUC values as effect sizes, and by comparing sensitivity and specificity values.

### Multi-biomarker Panels—The Better Choice

Overall, multi-biomarker panels are significantly more robust than single biomarkers alone, and in the context of PDAC, this holds true for both CA19-9 and novel biomarker subgroups, as well as across different patient control cohorts. This review shows extensive evidence, both graphically and statistically, that panels of more than one biomarker tend to perform better than single biomarkers alone for the diagnosis of PDAC. Furthermore, it was evident when eliminating confounding variables by subdividing the data into different groups, using variables such as biomarker type (CA19-9 or novel) or patient control cohort examined (healthy, benign, or mixed), that this result is robust and prevails throughout multiple subgroup analyses. Importantly, the inclusion of studies that do not specify PDAC did not greatly alter the results of the meta-analysis, suggesting that the cohorts within these studies are similar to those included in the original analysis. While there were many single biomarkers reported in the included studies with impressive efficacy, the results of the meta-analysis indicate that on the whole, multi-biomarker panels produce the most robust diagnostic performance. This information is crucial for future studies, as it suggests that researchers should focus their efforts on the identification of multiple biomarkers, rather than attempting to isolate one single biomarker. It is important to note also, that the creation of a multi-biomarker panel is not as straightforward as it may seem, and when dealing with multiple levels of patient data and consequently different cutoffs for individual biomarkers, care must be taken to ensure the desired sensitivity and specificity of the panel as a whole. Integration of these data will allow researchers the flexibility to tailor their panel to certain conditions, and determine individual cutoffs based on the needs of the test (5). Computational approaches, such as machine learning, have shown utility in this context and could provide future research with a more streamlined approach to multi-biomarker panel generation (29). One caveat to this, however, is the integration of multi-omic data, which would require cautious consideration of the various unit measurements involved and, in each case, careful control of confounding variables and potential artefacts of experimental design (30). In any case, whether multi-omic or single-omic, the thoughtful generation of highly sensitive and specific multi-biomarker panels should arguably be the primary aim of future studies hoping to identify novel diagnostic biomarkers for PDAC.

### CA19-9 and its Role as the Current Clinical Standard Biomarker

As CA19-9 is regarded as the current standard biomarker for pancreatic cancer diagnosis, the data were separated into two groups, those including CA19-9 and those without (novel biomarkers), to evaluate the performance of CA19-9 across all studies. For both subgroups of data, multi-biomarkers were shown to perform significantly better than single biomarkers alone. Indeed, we found that while CA19-9 is the “gold standard” for pancreatic diagnosis, the addition of some other biomarker to create a multi-biomarker panel with CA19-9 resulted in improved biomarker efficacy compared with CA19-9 alone. Furthermore, there was a substantial amount of variation between studies in the reported sensitivity and specificity values of CA19-9, and this is in keeping with current literature (31–34).

The results of the meta-analysis showed that the addition of CA19-9 to a multi-biomarker panel provided a clear improvement over novel biomarker panels that did not contain CA19-9. In addition, CA19-9 alone appears to have consistently outperformed novel single biomarkers. While CA19-9 may be the most commonly used biomarker for diagnosis of PDAC in patients with pancreatic cancer, elevated expression has been shown in various benign conditions such as pancreatitis, which contributes to its nonspecificity for PDAC (34). Given the similarities that are often observed between benign pancreatic patient blood and pancreatic cancer patient blood, it follows therefore that a biomarker may have a diminished ability to distinguish benign cohorts from those with cancer when compared with healthy cohorts. To ensure the differences being observed were not a result of the patient cohorts being evaluated by different studies, we separated the data further based on the patient cohorts distinguished from PDAC: PDAC versus healthy, PDAC versus benign, and PDAC versus mixed. When separated based on patient control cohorts, the improved ability of CA19-9 biomarkers over novel biomarkers to diagnose PDAC is clear.

It is also demonstrated here that the efficacy of a biomarker or biomarker panel to diagnose PDAC, whether including CA19-9 or a novel biomarker, is dependent on the reference cohort in question. Both CA19-9 and novel biomarkers exhibit improved ability to distinguish patients with PDAC from healthy controls when compared with both benign and mixed cohorts, with CA19-9 multi-biomarker panels producing the best diagnostic performance across all cohorts. There is considerable variation across pooled AUC values for CA19-9 biomarkers and novel biomarkers in the mixed patient cohort setting, demonstrating the increased difficulty faced in this control cohort. As a result of the recognized poor specificity of CA19-9, the lack of a standardized detection method (10), and the variation in cutoff levels being used for PDAC diagnosis across the studies (35–37), in clinical practice CA19-9 is rarely relied upon for diagnostic purposes, despite being FDA approved for this indication. More often it is used to support a diagnosis, based on appropriate imaging and/ or biopsy, or in staging with Immuno-PET imaging (38), as a biomarker of recurrence (9, 39), or as a biomarker of tumor resectability (40, 41). In fact, the results of this meta-analysis provide a strong argument in favor of the inclusion of CA19-9 when evaluating a new biomarker panel, while exercising caution given the variation in results obtained across different studies, and the reduced potential of CA19-9 in certain benign conditions. The role of CA19-9 in pancreatic cancer diagnosis, therefore, seems reliant on the identification of a robust multi-biomarker panel that can adequately control for the inherent defects of the biomarker.

### Multi-omics in Biomarker Identification

While it is evident from the results of this review that multi-biomarker panels are the most robust biomarker type, it remains to be seen which biological factors produce the most robust biomarkers. A major limitation of this review results from the lack of diversity seen in the “omic” compartments (genomics, proteomics, etc.) of biomarkers. Indeed, proteomic biomarkers make up the vast majority of biomarkers evaluated across all studies in this review, making comparisons between proteomic biomarkers and other omic compartments difficult. We can see, however, that the combination of different omic compartments with CA19-9 (proteomics) did result in high sensitivity and specificity values, though the number of studies examining multi-omic biomarker panels is too low to see any distinct difference. By examining the biomarkers that fall into the 90th percentile for both sensitivity and specificity, it is evident that while proteomic biomarkers may represent the majority, they do not solely comprise the top biomarkers. Indeed, nearly as many of these “top” biomarkers were transcriptomic in nature, highlighting the importance of examining different biological compartments for the discovery of robust biomarkers. Furthermore, while instances where multiple omic compartments were integrated to form a panel were uncommon among the papers included in this review, one multi-omic biomarker panel was among the 90th percentile biomarkers. A 4-biomarker panel containing CA19-9 and three metabolites was among those with the highest sensitivity and specificity, demonstrating the potential for such multi-omic biomarker panels in this context. While it is not within the scope of this review to evaluate whether multi-omic panels produce better results than single-omic panels, current trends in biomarker discovery are leaning toward multi-omic data integration (5, 42, 43). The evaluation of multiple biological compartments to give a comprehensive overview of disease, and subsequently the generation of a robust panel that encompasses the complexity of that disease is an appealing concept that has much potential in this context and requires further elucidation.

### Promising Novel Biomarkers for Pancreatic Cancer Diagnosis

Systematic reviews are uniquely poised to identify trends in the literature that may otherwise go unnoticed. Importantly, this systematic review allowed for the identification of 13 novel biomarkers that have been repeatedly examined as blood-based biomarkers for PDAC diagnosis across multiple studies, and show promise both alone and as part of a multi-biomarker panel. While again, the majority of these biomarkers are proteins, the transcriptomic biomarker TIMP-1 emerged as the most frequently assessed novel biomarker. Though it showed poor mean sensitivity and specificity alone, TIMP-1 performed well as part of a panel with improved mean sensitivity, specificity, and AUC values. This is further evident from its appearance among the 90th percentile biomarkers, where it achieved high sensitivity and specificity as part of an 8-biomarker panel of extracellular vesicle long RNAs. Because of its association with cell survival, cell growth, and tumorigenesis, the TIMP-1 protein has been investigated as a potential biomarker, both alone and as part of a panel, in several other cancer types such as gastric (44, 45), colorectal (46–48), and breast (49, 50). This association, however, could be the reason for TIMP-1s poor utility alone in PDAC diagnosis. Indeed, TIMP-1 protein performance as a blood-based biomarker in PDAC has been shown to be impaired in patients with jaundice (51), though not in patients with chronic pancreatitis (52, 53). Furthermore, TIMP-1 expression is known to be increased in patients with, and at an increased risk of developing, type 2 diabetes (54, 55), as well as in obese patients (56). This evidence suggests that the utility of TIMP-1 in PDAC diagnosis is promising, and may lie in its addition to a biomarker panel rather than its use alone due to its impairment in patients with several benign conditions. Interestingly, TIMP-1 has been examined alongside another promising novel biomarker, inflammatory protein leucine-rich-alpha-2-glycoprotein 1 (LRG1). LRG1 has been shown to promote angiogenesis and regulate tumorigenesis, and is a promising biomarker candidate for several other cancer types (57). Indeed, a plasma-based panel of TIMP-1, LRG1, and CA19-9 discriminated PDAC from healthy controls with improved accuracy compared with CA19-9 alone (58). LRG1 has also been evaluated in plasma alongside TTR and CA19-9, where this panel exceeded the accuracy of CA19-9 alone by over 10% in its ability to discriminate PDAC from benign controls and other cancers (59). While LRG1 shows promise as part of a panel for PDAC diagnosis, it has poor mean sensitivity and specificity alone, and there is a lack of research into its performance in control cohorts with various benign conditions.

Conversely, cancer antigen 125 (CA125), carcinoembryonic antigen (CEA), and carbohydrate antigen 242 (CA242) have been evaluated extensively in pancreatic cancer and as such there is a plethora of research on these biomarkers. CEA is an established and widely used tumor biomarker that is known to be increased in several cancers such as colorectal (60), breast (61), and lung (62). While CEA levels are currently measured for PDAC diagnosis in some clinical settings, it is not FDA approved for PDAC diagnosis and its utility and accuracy remains limited, with a 2018 systematic review and meta-analysis reporting CEA to be inferior to CA19-9 (63). CEA levels are also known to be elevated in patients with chronic pancreatitis, with serum CEA being unable to distinguish patients with PDAC from those with chronic pancreatitis (64). Indeed, in this review, we show that CEA alone exhibits poor diagnostic performance across included studies, with improved results being obtained when CEA is examined as part of a panel. CA125 is a known biomarker for ovarian cancer, which has been shown to have superior performance to CEA for PDAC diagnosis (65, 66). It has also produced higher mean sensitivity, specificity, and AUC values than CEA across the studies included here. Furthermore, a 2017 systematic review and meta-analysis showed that a CA125-based diagnostic panel for PDAC was superior to CA125 or CA19-9 alone (67). Similar to CEA, CA242 has also been extensively evaluated for PDAC diagnosis, with serum CA242 levels having been shown to positively correlate with CA19-9 levels (68). CA242 has also been demonstrated to have better diagnostic performance than CEA, with mean sensitivity, specificity and AUC values in this study being higher for CA242 than CEA (69). Unfortunately, CA242 is known to be elevated in the blood of patients with type 2 diabetes, and as such, has limited utility alone for PDAC diagnosis, despite exhibiting higher specificity than CA19-9, CEA, and CA125 (65, 70). While none of these biomarkers have stood out on their own as having utility across all patient cohorts, they are frequently examined as part of biomarker panels with other novel biomarkers. Laminin subunit gamma-2 (LAMC2), for example, is a promising new biomarker which was examined in a large-scale study of over 400 patients across three continents, where it was elevated in pancreatic cancer serum compared with controls and demonstrated a sensitivity that was comparable with CA19-9 (71, 72). Furthermore, a serum-based panel of LAMC2 with both CA19-9 and CA125 has been shown to produce accurate discrimination of PDAC from benign controls (33). These studies provide promising results for LAMC2 as a potential diagnostic biomarker both alone and as part of a panel, though the breadth of research is limited at this time, with no results for mean sensitivity or specificity being obtained in this review. Another promising biomarker that has produced results similar to CA19-9 is MUC5AC. Serum MUC5AC levels have been shown to be increased in patients with PDAC compared with both benign and chronic pancreatitis cohorts, with MUC5AC performing on par with CA19-9, though again, the combination of the two produced the best results (73, 74). Interestingly, the measurement of the CA19-9 antigen on circulating MUC5AC proteins showed promise in a study comprising over 500 patients from three different institutions, where both the sensitivity and specificity of the biomarker were improved by this method compared with measuring just CA19-9 alone (75). While the initial research on MUC5AC shows favorable results, there are few papers examining the capability of MUC5AC as part a multi-biomarker panel.

The addition of CA19-9 to some novel biomarker is a trend across most studies aimed at identifying diagnostic biomarkers for PDAC, with results generally reporting an improved result from the panel compared to individual biomarkers alone. Studies examining the potential of ALB in this setting are no different, with the vast majority of entries for ALB in this review originating from multi-biomarker panels. Indeed, a 5-biomarker panel containing ALB and CA19-9 produced improved diagnostic capabilities compared with CA19-9 alone (76). Similarly, the combination of ALB with CA19-9 and IGF-1 also performed better than CA19-9 at distinguishing PDAC from chronic pancreatitis (64). Interleukin-8 (IL-8), a proinflammatory cytokine, has also shown limited utility alone but appears to achieve reasonable results when included in a panel. Serum IL-8 has been shown to be higher in patients with pancreatic cancer than controls; however, the mean AUC value for IL-8 alone is poor and improved when included in a panel with other biomarkers (77–79). This is also the case for THBS2, which produces modest discrimination alone, and is significantly improved when examined alongside CA19-9 (80, 81). Conversely, c-reactive protein (CRP), a biomarker of inflammation, has shown limited utility as part of a panel, where the panel showed no improvement with the addition of CRP (64). A 2020 study also showed a significant difference in CRP levels between PDAC and normal controls; however, after running extensive statistical tests on all candidate biomarkers it was not included in the final panel of six biomarkers (82). While CRP is increased in patients with PDAC compared with controls, it is also elevated in patients with moderate and severe pancreatitis (83). Moreover, as CRP is derived from the liver, it is substantially influenced by the presence of jaundice making it unreliable in patients with this comorbidity (64). Interestingly, several studied have examined these more “unreliable” candidates together, and obtained promising results. A 4-biomarker panel with CA19-9, CRP, and IL-8 demonstrated good discrimination of PDAC from controls (84). While a 2014 study showed that a panel consisting of ALB, CA19-9, CRP, and IL-8 had the highest diagnostic value for distinguishing PDAC from controls, with this panel proving to be effective in identifying other cancers, such as breast, cervical, colorectal, prostate, and lung (84). These studies highlight the utility of all of these biomarkers together, rather than independently.

Finally, two miRNA emerged as the most frequently examined across the studies included in this review, miR-21 and miR-483. MiR-21 levels in the circulation have been shown to be higher in PDAC compared with healthy controls, and are also associated with advanced stage, metastasis, and shorter survival (85, 86). However, miR-21 shows poor discriminatory ability between IPMN and PDAC, suggesting the involvement of miR-21 in an early step of pancreatic tumorigenesis (85). Indeed, the ability of miR-21 to distinguish PDAC from controls was overshadowed by several other miRNA in a 2019 study, such as miR-33a and miR-320a, which outperformed miR-21 in combination, thus excluding miR-21 from the final panel (87). Overexpression of miR-483 is also thought to be an early event in PDAC progression, having been shown to be present in premalignant pancreatic cystic lesions and early-stage disease (88). A 2016 large-scale miRNA study with over 400 patients with PDAC showed that serum miR-483 expression was significantly increased in patient with PDAC compared with both benign and healthy controls together (89). Unfortunately, there is a lack a research into the diagnostic potential of these individual miRNAs, as most studies focus on the large-scale screening of miRNAs and utilize complex modeling to narrow down their validation cohort to the most statistically relevant biomarkers.

On closer examination of the literature around these frequently examined biomarkers, it is clear that no one biomarker produces highly accurate diagnostic results alone. Indeed, the evidence would suggest that the primary utility of all of these biomarkers can be found in their use within multi-biomarker panels. While individually each of these biomarkers has their limitations, it is evident that when put together they can account for the weaknesses of the others to improve the end results. Furthermore, the addition of CA19-9 stands out as a clear prerequisite for the design of future multi-biomarker panels. These novel candidates provide a glimpse into the promising future of PDAC diagnostic biomarker discovery, though they remain to be examined within cohorts of patients with various underlying conditions and comorbidities that may influence their performance. Importantly, while blood-based biomarkers in the PDAC setting are likely to be used primarily as companion diagnostics, several of these biomarkers may also prove useful in the risk stratification of pancreatic patients with underlying conditions, given their dysregulation across certain control cohorts as outlined in this study.

### The State of Current Pancreatic Cancer Research

This review highlights the variability in data quality and study design across pancreatic cancer research. Here, we have interrogated studies which employ biomarkers for the diagnosis of PDAC, identifying many studies that fail to provide sufficient information regarding their patient cohorts, their experimental design or their index test of interest. A substantial number of papers fail to report on the subtype of pancreatic cancer examined, simply conflating all subtypes as pancreatic cancer. For the purposes of the meta-analysis, papers that do not specify PDAC as the subtype of interest were excluded so as to reduce confounding variables. However, this lack of detail is a major flaw within many pancreatic cancer studies, where the specific subtype examined should always be clearly indicated.

Furthermore, almost half of the included biomarker entries did not have information regarding the reference standard used to diagnose patients with PDAC. In these cases, it was unclear whether all patients in this cohort had been diagnosed using the same reference standard or not, resulting in high levels of bias amongst these papers. A third of the biomarkers examined had no details attributed to them regarding the stage details of the PDAC cohort, with reporting of sex and age breakdowns in this cohort also poor. Control cohorts had similar issues, with high numbers of biomarkers also lacking sex and age information.

Unfortunately, the number of studies examining arguably the most clinically relevant control cohort (mixed) is extremely low compared with healthy alone and benign alone. While this review has identified many studies evaluating various types of biomarkers for PDAC diagnosis, the lack of studies conducted in clinically relevant cohorts may be the reason for the unfortunate lack of biomarkers currently in clinical use. Blinding of studies was also extremely poor, with very few opting to adopt this strategy for biomarker identification. This has further contributed to the high levels of bias observed across the studies included in this review.

Finally, evaluation of biomarker efficacy was extremely flawed in some cases, with a substantial number of biomarkers being attributed only with a *P* value and no qualitative assessment (e.g., AUC or sensitivity) of the biomarker. Overall, huge flaws exist in current pancreatic cancer research in the context of identification of biomarkers for PDAC diagnosis. High levels of bias can be seen in many studies, with missing or unclear information regarding key study design points further compounding these issues. These are major flaws which recur again and again in the literature and could be contributing to the lack of repeated examination of high performing biomarkers in follow-up studies and could subsequently be responsible for the poor progress seen in this field in recent years.

### Limitations of This Systematic Review and Meta-analysis

As modern vernaculars regard pancreatic cancer and its PDAC subtype to be synonymous, any paper that did not specify an alternative subtype of pancreatic cancer was included in the extraction stage of this study and assumed to be PDAC. While a small minority of the total included studies make up this population, it is important to note that the inclusion of these data may not be appropriate in some cases as PDAC may not have been the subtype of pancreatic cancer examined. CA19-9 is highlighted in this review as the current FDA-approved biomarker for PDAC diagnosis; however, CA19-9 cut-off values were not standardized across all studies included in the review. As such, all CA19-9 entries may not have resulted from the same cut-off value and it may not be appropriate to compare them directly, as changes in CA19-9 cut-off values have been demonstrated to improve biomarker robustness (33). A major caveat of this review, which results from the nature of the data extraction, is that certain biomarkers or biomarker panels may arise several times from a single study, having been examined in multiple patient cohorts within that study, for example, in the context of model training and validation. Unfortunately, as in many studies, there can be overlap between the patients recruited for the training and validation cohorts, resulting in repeated sampling from the same patients. The within-study variance has been controlled for in the multivariate meta-analysis; however, repeated sampling from the same patients was not accounted for and may introduce a level of bias toward some biomarkers. Furthermore, in many instances, studies have opted to evaluate several single biomarkers and subsequently combine these biomarkers to form a multi-biomarker panel. Some multi-biomarker panels have also been examined in some studies both alone and with the addition of CA19-9 to the panel. Possible bias due to repeated entries is an important limitation of this study, which could not be avoided due to the nature of current research papers and study designs. Importantly, while the QUADAS-2 tool that was used to assess study quality and RoB has been used previously for similar systematic reviews of diagnostic biomarkers (90), there may be other forms of bias introduced by these studies that were not accounted for in this assessment.

## Conclusions

In summary, blood-based multi-biomarker panels for the diagnosis of PDAC exhibit superior performance in comparison with single biomarkers, in both CA19-9–containing biomarkers and novel biomarkers, and across all patient control cohorts. CA19-9 shows little utility alone, as it is less effective in mixed control cohorts, though when used in combination with a panel of multiple biomarkers these CA19-9–containing panels produce a better diagnostic performance than novel multi-biomarker panels. These results suggest that future biomarker studies for PDAC diagnosis should focus on the identification of a multi-biomarker panel which includes CA19-9, while drawing from the pool of promising novel biomarkers that have been identified and examined across several different studies. This will allow for better use of the breadth of knowledge that has been accumulated over decades of research and save valuable time and resources as studies steer away from large-scale fishing expeditions, and move toward more focused and specialized research with appropriate blinding and comprehensive experimental design.

## Supplementary Material

Supplementary Material S1 PRISMA 2020 checklist

Supplementary Material S2 PRISMA flow chart

Supplementary Material S3 Individualised search strategy

Supplementary Material S4 Inclusion exclusion criteria

Supplementary Material S5 Complete list of extracted data fields

Supplementary Material S6 QUADAS-2

Supplementary Material S7 Complete extracted dataset

Supplementary Material S8 Figure S8 panels A-E
