# Supplementary figures and images for: Diagnostic Accuracy of Blood-based Biomarkers for Pancreatic Cancer: A Systematic Review and Meta-analysis
Source: Cancer Res Commun. 2022 Oct 20;2(10):1229–43. doi: 10.1158/2767-9764.CRC-22-0190 (PMC10035398; doi:10.1158/2767-9764.CRC-22-0190)

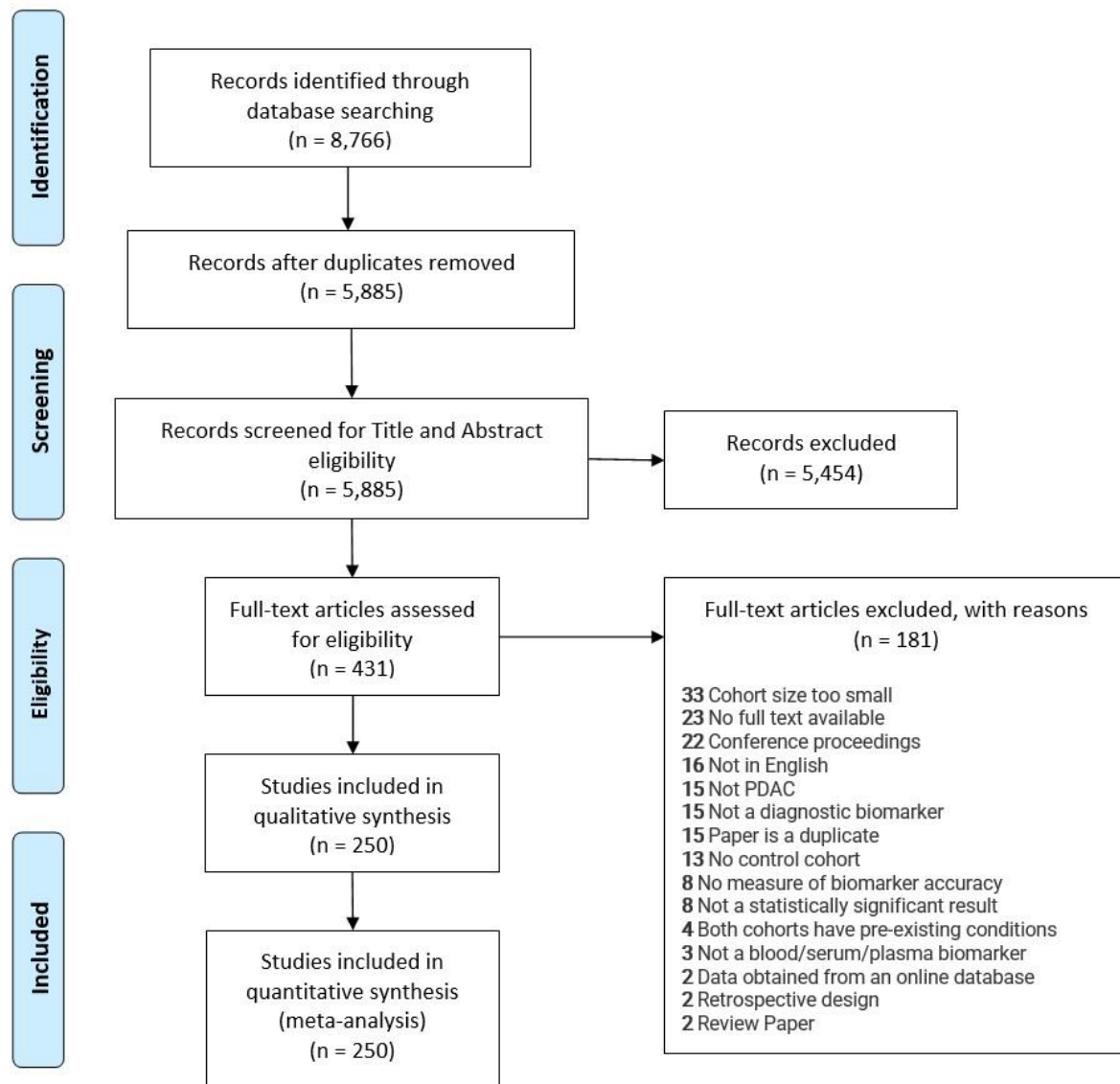

**Supplementary Material S2. PRISMA flow diagram of record selection process.**

Supplement: Supplementary Material S2 — PRISMA flow chart [file crc-22-0190-s02.pdf]
