# Supplementary material for: Diagnostic Accuracy of Blood-based Biomarkers for Pancreatic Cancer: A Systematic Review and Meta-analysis
Source: Cancer Res Commun. 2022 Oct 20;2(10):1229–43. doi: 10.1158/2767-9764.CRC-22-0190 (PMC10035398; doi:10.1158/2767-9764.CRC-22-0190)
Supplement: Supplementary Material S3 — Individualised search strategy [file crc-22-0190-s03.pdf]

Supplementary Material S3. Individualised search strategies for academic databases.

Database	Search strategy
EMBASE	'pancreas adenocarcinoma'/exp<br>(pancrea* NEAR/3 adenocarcinoma*):ti,ab<br>PDAC:ti,ab<br>#1 OR #2 OR #3<br>'biological marker'/exp OR 'tumor marker'/exp<br>(Biomarker* OR ‘biological indicator*’):ti,ab<br>((biological OR serum OR Immunologic OR diagnostic OR tumo?r OR cancer) NEAR/2 (marker* OR biomarker* OR bio-marker*)):ti,ab<br>#5 OR #6 OR #7<br>'diagnosis'/exp<br>Diagnos*:ti,ab<br>#9 OR #10<br>#4 AND #8 AND #11<br>'editorial'/exp OR 'erratum'/de OR 'letter'/exp OR 'conference abstract':it OR 'conference review':it<br>#12 NOT #13
Medline	Carcinoma, Pancreatic Ductal/ OR (exp Pancreatic Neoplasms/ AND exp *Adenocarcinoma/)<br>(pancrea* adj3 adenocarcinoma*).ti,ab.<br>PDAC.ti,ab.<br>or/1-3<br>exp Biomarkers/<br>(Biomarker* OR biological indicator*).ti,ab.<br>((biological OR serum OR Immunologic OR diagnostic OR tumo?r OR cancer) adj2 (marker* OR biomarker* OR bio-marker*)).ti,ab.<br>or/5-7<br>exp Diagnosis/<br>diagnos*.ti,ab.<br>or/9-10<br>4 AND 8 AND 11
Web of Science	TS =(((pancrea* NEAR/2 adenocarcinoma*) OR PDAC) AND ((Biomarker* OR “biological indicator*”) OR ((biological OR serum OR Immunologic OR diagnostic OR tumo?r OR cancer) NEAR/2 (marker* OR biomarker* OR bio-marker*))) AND Diagnos*)
