# Supplementary material for: Diagnostic Accuracy of Blood-based Biomarkers for Pancreatic Cancer: A Systematic Review and Meta-analysis
Source: Cancer Res Commun. 2022 Oct 20;2(10):1229–43. doi: 10.1158/2767-9764.CRC-22-0190 (PMC10035398; doi:10.1158/2767-9764.CRC-22-0190)
Supplement: Supplementary Material S4 — Inclusion exclusion criteria [file crc-22-0190-s04.pdf]

Supplementary Material S4. Inclusion and exclusion criteria.

Inclusion Criteria	Exclusion Criteria
• Human, primary case-control study.<br>• Diagnostic cohort studies.<br>• Primary pancreatic ductal adenocarcinoma (PDAC) of any stage.<br>• Diagnostic biomarker for PDAC.<br>• Must have a control cohort of healthy and/or benign patients.<br>• Minimum 15 patients per cohort.<br>• Must report some summary statistic for the performance of the biomarker.<br>• Must be a whole blood/serum/plasma biomarker.<br>• Must report a significant result.	• Narrative or systematic review.<br>• Patient data is obtained from online database such as The Cancer Genome Atlas (TCGA).<br>• All participants, PDAC and controls, have pre-existing conditions that may confound the index test, e.g., diabetes or pancreatitis.<br>• Studies with a retrospective design, where investigators chose participants based on a review of case notes/ archival information.<br>• Case reports or series.<br>• Conference proceedings, where a full text is not available.
