# Supplementary material for: Diagnostic Accuracy of Blood-based Biomarkers for Pancreatic Cancer: A Systematic Review and Meta-analysis
Source: Cancer Res Commun. 2022 Oct 20;2(10):1229–43. doi: 10.1158/2767-9764.CRC-22-0190 (PMC10035398; doi:10.1158/2767-9764.CRC-22-0190)
Supplement: Supplementary Material S5 — Complete list of extracted data fields [file crc-22-0190-s05.pdf]

Supplementary Material S5. Complete list of extracted data fields.

**Paper Information**	○ Covidence study ID number<br>○ Paper title<br>○ Corresponding author name<br>○ Corresponding author email address<br>○ Country in which study was conducted<br>○ Study funding source<br>○ Conflicts of interest<br>○ Ethical approval
**Study Design**	○ Study design (prospective/retrospective)<br>○ Fluid type (serum/plasma/whole blood)<br>○ Time of blood draw relative to treatment/FNA /surgery<br>○ Start date<br>○ End date<br>○ PDAC specified<br>○ Paper examined more than one biomarker<br>○ Test platform<br>○ Testing/training /validation cohort<br>○ Blinded
**Biomarker details**	○ Biomarker(s) name<br>○ Number of biomarkers<br>○ Biological Properties<br>○ Single biomarker or multi-biomarker panel<br>○ Number of omics compartments for each biomarker/panel<br>○ Whether CA19-9 or novel
**Patient Cohorts**	○ PDAC / Healthy / Benign<br>○ Details of how control cohort was chosen<br>○ Details of benign conditions and breakdown of numbers<br>○ Number of patients<br>○ Males and Females<br>○ Age range/mean/median<br>○ Method of PDAC confirmation<br>○ Stage details for PDAC<br>○ Whether patients received treatment prior to blood draw<br>○ Treatment details
**Statistical details**	○ Confounding factors of note<br>○ Statistical analyses used<br>○ Sensitivity/Specificity<br>○ AUC/ROC<br>○ P-value<br>○ Negative predictive value/positive predictive value
**Extra Notes**	○ Name of reviewer who extracted data<br>○ Additional notes
