# Supplementary material for: Diagnostic Accuracy of Blood-based Biomarkers for Pancreatic Cancer: A Systematic Review and Meta-analysis
Source: Cancer Res Commun. 2022 Oct 20;2(10):1229–43. doi: 10.1158/2767-9764.CRC-22-0190 (PMC10035398; doi:10.1158/2767-9764.CRC-22-0190)
Supplement: Supplementary Material S6 — QUADAS-2 [file crc-22-0190-s06.pdf]

Supplementary Material S6. QUADAS-2 quality and risk of bias assessment questions.

<u>**Domain 1 – Patient Selection**</u><br>**Risk of Bias**<br><br>Q1. Was a consecutive or random sample of patients enrolled?<br>Q2. Did the study avoid inappropriate exclusions?<br>Q3. Was a 'two-gate' design avoided?<br><br>***Could the selection of patients have introduced bias?***	 <br><br>Yes / No / Unclear<br>Yes / No / Unclear<br>Yes / No / Unclear<br><br>High / Low / Unclear
<u>**Domain 1 – Patient Selection**</u><br>**Concerns about applicability**<br><br>***Are there concerns that the included patients do not match the review question?***	 <br><br>High / Low / Unclear
<u>**Domain 2 – Index Test**</u><br>**Risk of Bias**<br><br>Q1. Were the index test results interpreted without knowledge of the results of the reference standard?<br>Q2. If a threshold was used, was it pre-specified?<br><br>***Could the conduct or interpretation of the index test have introduced bias?***	 <br><br>Yes / No / Unclear<br><br>Yes / No / Unclear<br><br>High / Low / Unclear
<u>**Domain 2 – Index Test**</u><br>**Concerns about applicability**<br><br>***Are there concerns that the index test, its conduct, or interpretation differ from the review question?***	 <br><br>High / Low / Unclear
<u>**Domain 3 – Reference Standard**</u><br>**Risk of Bias**<br><br>Q1. Is the reference standard likely To correctly classify the target condition?<br>Q2. Were the reference standard results interpreted without knowledge of the results of the index tests?<br><br>***Could the reference standard, its conduct, or its interpretation have introduced bias?***	 <br><br>Yes / No / Unclear<br>Yes / No / Unclear<br><br><br>High / Low / Unclear
<u>**Domain 3 – Reference Standard**</u><br>**Concerns about applicability**<br><br>***Are there concerns that the target condition as defined by the reference standard does not match the question?***	 <br><br>High / Low / Unclear
<u>**Domain 4 – Flow and Timing**</u><br>**Risk of Bias**<br><br>Q1. Was there an appropriate interval between index test (sample collection) and reference standard?<br>Q2. Did all patients receive the same reference standard?<br>Q3. Were all patients included in the analysis?<br><br>***Could the patient flow have introduced bias?***	 <br><br>Yes / No / Unclear<br><br>Yes / No / Unclear<br>Yes / No / Unclear<br><br>High / Low / Unclear
