# Supplementary material for: Diagnostic Accuracy of Blood-based Biomarkers for Pancreatic Cancer: A Systematic Review and Meta-analysis
Source: Cancer Res Commun. 2022 Oct 20;2(10):1229–43. doi: 10.1158/2767-9764.CRC-22-0190 (PMC10035398; doi:10.1158/2767-9764.CRC-22-0190)
Supplement: Supplementary Material S8 — Figure S8 panels A-E [file crc-22-0190-s08.pdf]

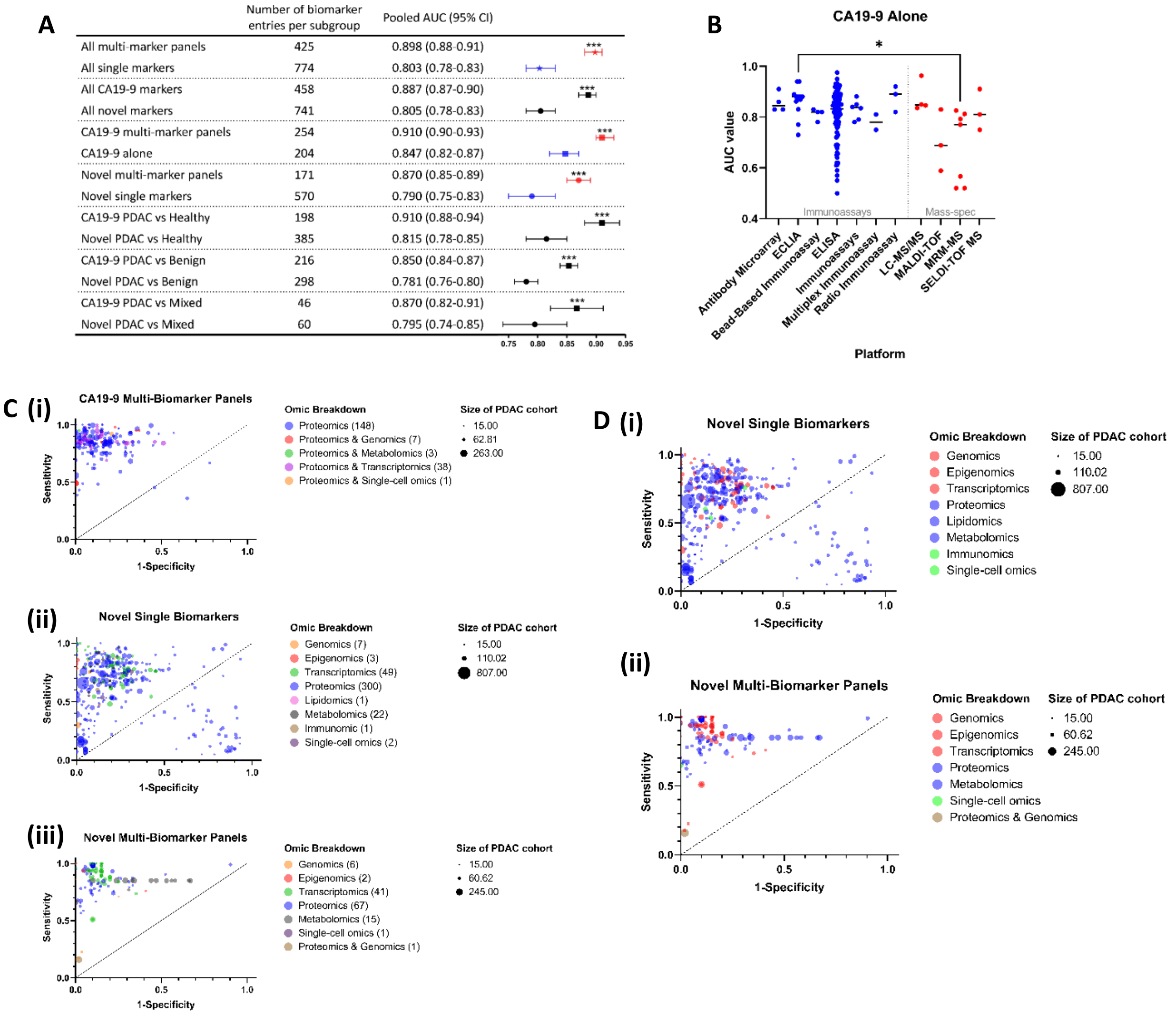

E

Single or multi	First author (year)	Study design	Fluid Type	CA19-9 or Novel	Biomarker(s)	Patient Selection: Risk of Bias	Patient Selection: Concerns about applicability	Index Test: Risk of Bias	Index Test: Concerns about applicability	Reference Standard: Risk of Bias	Reference Standard: Concerns about applicability	Flow and Timing: Risk of Bias
Multi-marker panels	Saito (1993)	Unsure	Serum	CA19-9	9-marker panel[1]	?	✓	?	✓	✓	✓	?
	Zhang (2020)	Unsure	Serum	CA19-9	4-marker panel[2]	?	✓	?	✓	?	✓	?
	Yanagisawa (2012)	Prospective	Plasma	Novel	7-protein signature[3]	✗	✓	?	✓	✓	✓	✓
	Gao (2012)	Prospective	Serum	Novel	4-peak panel[4]	✗	✓	✓	✓	✓	✓	✓
	Yu (2019)	Retrospective	Plasma	Novel	8-exLR signature[5]	?	✓	?	✓	?	✓	?
	Le Calvez-Kelm (2016)	Prospective	Plasma	Novel	cfDNA KRAS mutations[6]	✗	✗	✗	✓	?	✗	?
	Le Calvez-Kelm (2016)	Prospective	Plasma	Novel	cfDNA KRAS mutations[7]	✗	✗	✗	✓	?	✗	?
Single markers	Di Gangi (2016)	Prospective	Serum	Novel	Palmitic Acid	✗	✓	✓	✓	✓	✓	✓
	Kahlert (2014)	Unsure	Serum	Novel	MMP-12	?	✓	?	✓	?	✓	✗
	Kahlert (2014)	Unsure	Serum	Novel	MMP-12	?	✓	?	✓	?	✓	✗
	Wlodarczyk (2020)	Prospective	Serum	Novel	IGF-1	✗	✗	✗	✓	✗	✓	✗
	Hussein (2017)	Prospective	Serum	Novel	miR-642b-3p	✗	✗	✗	✓	✗	✓	✓
	Hussein (2017)	Prospective	Serum	Novel	miR-885-5p	✗	✗	✗	✓	✗	✓	✓
	Cote (2014)	Prospective	Plasma	Novel	miR-10b	✗	✗	✗	✓	?	✗	?
	Cote (2014)	Prospective	Plasma	Novel	miR-106b	✗	✗	✗	✓	?	✗	?

Supplementary Material S8. (A) Summary of multivariate three-level meta-analysis with subgroup moderators including all 250 studies. **(B)** Evaluation of platform-to-platform variation in CA19-9 detection ($p<0.05$, Kruskal-Wallis test with multiple comparisons). **(C)** Representative ROC plot showing the extracted sensitivity and 1-specificity for (i) CA19-9 multi-marker panels, (ii) novel single markers, and (iii) novel multi-marker panels. (Figures inside the brackets give exact numbers of markers from each omic compartment(s)). **(D)** Representative ROC plot showing the extracted sensitivity and 1-specificity for (i) novel single markers, and (ii) novel multi-marker panels. **(E)** Risk of Bias assessments for biomarkers included in the 90th percentile for sensitivity and specificity (Risk of bias assessment results are given for each biomarker as high (), low () and unclear ()).

[1]CA19-9, DUPAN-2, TPA, elastase-1, lipase, amylase, gamma-glutamyl transpeptidase, alkaline phosphatase and lactate dehydrogenase.
[2]CA19-9, docosahexanoic acid, lysoPC(14:0) and histidinyl-lysine.
[3]8562.3m/z, 8684.4m/z, 8765.1m/z, 9423.5m/z, 13761.5m/z, 14145.2m/z and 17250.8m/z.
[4]7775Da, 8567Da, 5362Da and 5344Da.
[5]FGA, KRT19, HIST1H2BK, ITIH2, MARCH2, CLDN1, MAL2 and TIMP1.
[6]cfDNA KRAS mutations at PDAC hotspot codons (12, 13, 61).
[7]cfDNA KRAS mutations at any screened codons reported in any cancer sites.
